# Editorial: Cell Physiology and Stress Adaptation of Crops

**DOI:** 10.3390/plants15172644

**Published:** 2026-08-28

**Authors:** Wei Jiang, Haitao Liu

**Affiliations:** 1 College of Advanced Agricultural Sciences (College of Plant Protection), Zhejiang A & F University, Hangzhou 311300, China; 2 College of Resources and Environment, Henan Agricultural University, Zhengzhou 450046, China

## 1. Introduction

**Figure 1:**
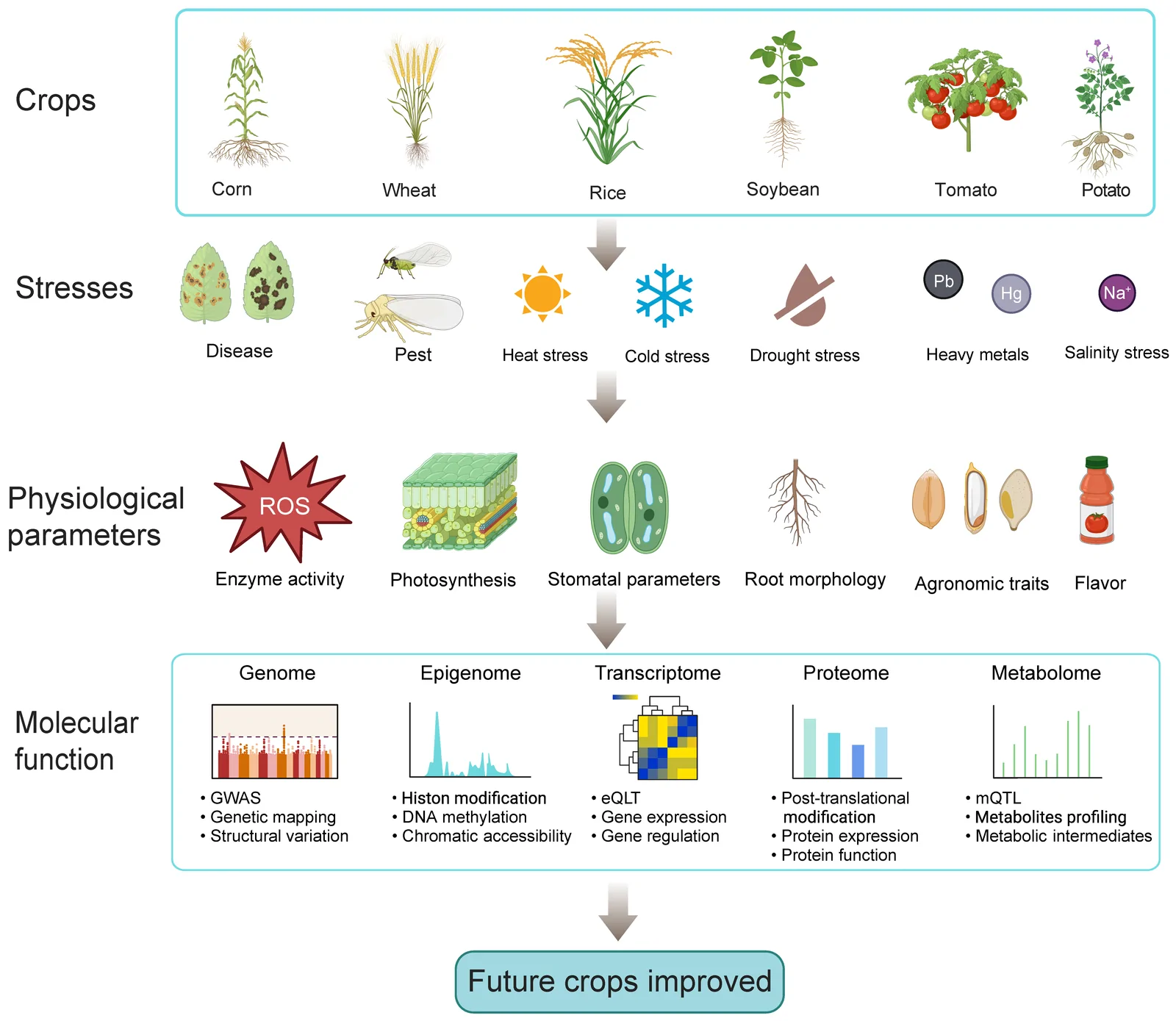
Research strategies for developing stress-tolerant crops. Under biotic and abiotic stresses, diverse crops exhibit significant physiological response differences. Omics such as genomics, epigenomics, transcriptomics, proteomics, and metabolomics might help us find key genes associated with stress tolerance traits. The figure was created using BioRender (https://app.biorender.com/).

Climate change has introduced various global-food-security-related challenges in recent years [1]. The increasing frequency and intensity of drought [2], hot and cold periods [3], salinity, flooding, and heavy-metal stresses [4] affect agricultural productivity worldwide [5]. Against this backdrop, it is critical to understand how crops perceive, transduce, and respond to these environmental stresses at the cellular and molecular levels [6] (Figure 1), as such knowledge will aid in the development of resilient crops that maintain productivity under adverse conditions [7]. The physiological and cellular mechanisms of plant stress responses encompass a complex network of signaling pathways [8], transcriptional reprogramming [9], metabolic adjustments [10], and cellular protection strategies [11]. This Special Issue of “Cell Physiology and Stress Adaptation of Crops” collects 23 original research articles and review papers (https://www.mdpi.com/journal/plants/special_issues/5RH2G2828D, accessed on 7 July 2026) that advance our understanding of these physiological and molecular mechanisms in multiple crops.

This Special Issue covers a wide array of abiotic stresses—including drought, salinity, heat, cold, heavy-metal toxicity, and nutrient deficiency—and explores beneficial microbial interactions and grafting techniques for enhancing stress tolerance. A shared theme in these studies is the importance of maintaining cellular homeostasis via integrated regulatory networks involving reactive oxygen species (ROS) [12], osmotic adjustment, phytohormone signaling [8,13], and transcriptional reprogramming [14]. Furthermore, several papers highlight how plants employ conserved molecular mechanisms such as antioxidant enzyme systems, osmolyte accumulation, and stress-responsive transcription factors, revealing species-specific adaptations and the potential for genetic improvement via marker-assisted breeding and transgenic approaches.

Interestingly, stress tolerance is governed by complex and interconnected regulatory cascades rather than individual pathways [13,15,16]. Multiple papers emphasize the roles of specific gene families in stress adaptation, including sulfate transporters (*SULTR*), sucrose non-fermenting-related kinases (*SnRKs*), the C3HC4-type RING zinc finger family (*C3HC4*), the abscisic-acid-stress-ripening family (*ASR*), heterotrimeric G-proteins, and aquaporins (*AQPs*). Moreover, several studies employ genome-wide identification and expression-profiling methods, revealing how these gene families have evolved and diversified across species to enable stress-specific responses [17,18]. The integration of multi-omics approaches in several papers provides systems-level insights into the molecular networks that coordinate physiological adjustments during stress exposure [19,20] (Figure 1).

The following summaries highlight the key findings and contributions of each paper in this collection. Many studies focus on the omics and physiological differences pertaining to plants in response to diverse stresses. For instance, Milanese et al. [14] examined how cold-tolerant bacteria isolated from alpine plants improve growth and mitigate cold stress in tomato (*Solanum lycopersicum*). They found that inoculation with *Chryseobacterium* sp. GRCS301 and *Pseudomonas* sp. GRCS202 lowered H_2_O_2_ content and triggered complex transcriptional reprogramming in tomato shoots. Bacterial inoculation increased the expression of genes involved in DNA replication, galactose metabolism, photosynthesis, ROS homeostasis, phytohormone signaling, and protein metabolism under cold stress. Their work demonstrates that beneficial microorganisms can enhance cold acclimation through coordinated transcriptional adjustments. Zhang et al. [19] employed temporal-resolution metabolomics to investigate polyphenolic dynamics during pepper graft healing. Analysis of 432 polyphenol metabolites across 12 time points revealed nine polyphenolics specifically responding to graft healing. This high-resolution temporal dataset provides a foundation for understanding the biochemical processes underlying graft union formation and the role of polyphenols in this critical developmental transition. Furthermore, Zhou et al. [20] integrated physiological measurements with multi-omics analyses (transcriptomics, proteomics, and metabolomics) to elucidate cadmium (Cd) response mechanisms in *Solanum americanum*, a potential phytoremediation candidate. Their integrated analysis revealed key proteins involved in phenylpropanoid biosynthesis (POD, PAL, F5H, COMT, and CAD) and photosynthesis pathways (GAPA, FBP, and FBA) that contribute to Cd tolerance, providing detailed mechanistic insights into Cd detoxification.

Additionally, Khamwong et al. [21] investigated drought-induced accumulation of bioactive compounds in *Clinacanthus nutans*, a medicinal plant with antioxidant and anti-inflammatory properties. Their integrative physiological and molecular analysis revealed that short-term drought (4–8 days) enhanced the accumulation of phenolic compounds, flavonoids, and triterpenoids such as schaftoside and lupeol, while prolonged stress reduced biosynthetic capacity. Molecular docking confirmed that there were favorable interactions between drought-induced metabolites and inflammation-related targets, suggesting controlled drought exposure is a viable cultivation strategy for enhancing medicinal compound production. Shen et al. [22] explored the effects of porcine blood polypeptide (PBP) priming on salt tolerance in wheat (*Triticum aestivum*). PBP-primed seedlings showed improved growth, enhanced root development, higher photosynthetic efficiency, and increased antioxidant enzyme activity (SOD, CAT, POD, and APX) relative to controls. Reduced levels of H_2_O_2_, O_2_^•−^, MDA, and electrolyte leakage indicated effective mitigation of oxidative stress. Principal component analysis identified antioxidant enzyme activity, osmotic regulation, and ROS accumulation as major factors in PBP-mediated stress responses. Han et al. [23] evaluated the effects of arbuscular mycorrhizal fungi (AMF) on wheat growth under low-potassium (K) stress. Inoculation with *Rhizophagus intraradices* significantly increased mycorrhizal colonization, biomass, ascorbic acid, and glutathione content while reducing malondialdehyde levels. K transporter gene expression was particularly enhanced under AMF combined with low-K stress, suggesting AMF primarily affects K uptake and transport in roots. This work supports the application of AMF as a sustainable agricultural strategy in low-K soils. In addition, Yang et al. [24] investigated the effects of drought stress at the booting stage on rice leaf physiology and yield across three varieties. Drought significantly decreased chlorophyll content, photosynthesis, and yield, with the HY73 variety showing the greatest drought tolerance. Yield reduction correlated with decreased grain number per panicle, seed-setting rates, and 1000-grain weight. Correlation analysis identified significant positive relationships between yield and photosynthetic parameters, emphasizing the importance of maintaining photosynthetic efficiency under drought. Overall, through combining multi-omics data, we may acquire systemic insights into the regulatory networks governing physiological adaptation to distinct stresses [25] (Figure 1).

Furthermore, evolutionary analysis demonstrates the conservation and divergence of different gene families in plants. For instance, Zhu et al. [17] performed genome-wide analysis of *SULTR* genes in plants, indicating the expansion of this family in gymnosperms and angiosperms. They also identified 16 *HvSULTR* genes and revealed that *HvSULTR2*/*4/5* levels increased after four days of heat stress in barley (*Hordeum vulgare*). Functional characterization revealed that HvSULTR11 localized to the plasma membrane and interacted with 14-3-3 proteins. Furthermore, haplotype analysis across 123 barley varieties provided candidate gene resources for enhancing stress tolerance in breeding programs. Shi et al. [26] characterized the Polycomb group (*PcG*) gene family in rice, identifying 15 *OsPcG* genes across 12 chromosomes. Transcriptome analysis under salt treatment identified *OsPcG5* as a key salt-responsive gene. Overexpression of *OsFIE2*, a PRC2 component, resulted in elevated H3K27me3 levels and abnormal plant height, linking epigenetic regulation to both development and stress responses. Overall, this work provides genetic resources for enhancing salt tolerance through epigenetic approaches. Lu et al. [27] characterized the *SnRK* gene family in *Taxus*, an ancient gymnosperm valued for paclitaxel production. They identified 19 *SnRK* genes and developed a PEG-mediated protoplast transient expression system to overcome experimental constraints in this non-model plant. *TaSnRK1.2* was identified as a hub integrating stress signals to mediate downstream transcription factor networks, establishing a foundation for functional studies on medicinal plants with long-lasting generations. Chen et al. [28] systematically compared *C3HC4* genes between salt-tolerant barley and salt-sensitive rice. They identified 123 *HvC3HC4* genes and 90 *OsC3HC4* genes, revealing that barley preferentially adopts an energy-conserving strategy that may contribute to its superior salt tolerance. This comparative genomic analysis offers insights into the evolutionary specialization of stress-responsive gene families and potential targets for improving salinity tolerance in cereals. Ren et al. [29] identified ten barley *ASR* genes and analyzed their expression under salt, osmotic, and abscisic acid (ABA) treatments. *HvASR2* and *HvASR3* were characterized as transcriptional repressors and activators, respectively. Cis-acting elements including *ABRE*, *MYB*, *ARE*, and *STRE* were identified in *HvASR* promoters. Furthermore, Wu et al. [30] characterized the chalcone isomerase (*CHI*) gene family in sweet potato (*Ipomoea batatas*), identifying five *IbCHI* genes. *IbCHI1* demonstrated CHI catalytic activity in transgenic calluses. Expression analysis revealed tissue-specific patterns and differential responses to abiotic stresses, with the catalytic genes *IbCHI1* and *IbCHI5* serving as primary stress responders, while the non-catalytic members *IbCHI3* and *IbCHI4* may fine-tune metabolic flux under stress conditions. Han et al. [18] identified eight heterotrimeric *G-protein* genes in barley and analyzed their expression under salt, drought, and waterlogging stress, revealing tissue-specific expression patterns and differential regulation in response to abiotic stresses. Their findings suggest that G-protein genes may contribute to barley’s salt tolerance mechanisms and serve as candidate genes for improving crop resilience.

Several studies also characterized gene functions in response to diverse types of stress. Cao et al. [31] functionally characterized *FvHsfB1a*, a class B heat stress transcription factor from woodland strawberry (*Fragaria vesca*). Ectopic expression of *FvHsfB1a* in Arabidopsis improved thermotolerance, resulting in higher germination and survival rates, enhanced proline and chlorophyll content, and reduced oxidative damage. The authors further demonstrated that *FvWRKY75* activates the *FvHsfB1a* promoter through W-box recognition, establishing a transcriptional cascade regulating heat stress responses. Zhu et al. [32] characterized *StMAPKK1*, a group D MAPKK in potato (*Solanum tuberosum*), and demonstrated its positive role in heat stress tolerance. Overexpression lines exhibited elevated antioxidant enzyme activity (APX, CAT, SOD, and POD), reduced oxidative damage, improved photosynthetic efficiency, and enhanced tuber yield under heat stress. RNAi-mediated suppression confirmed *StMAPKK1*’s essential role, indicating that it is a key genetic target for breeding heat-tolerant potato varieties. Li et al. [33] demonstrated that *FvWRKY75* positively regulates salt stress tolerance in strawberry by directly binding to the *FvCRK5* promoter through W-box elements. Ectopic expression of either *FvWRKY75* or *FvCRK5* in Arabidopsis enhanced salt tolerance through reducing ROS accumulation, increasing chlorophyll content, and enhancing antioxidant enzyme activity. This WRKY-CRK regulatory network constitutes a novel mechanism for salt stress adaptation in strawberry. Furthermore, Luo et al. [34] used CRISPR/Cas9 to generate an *abi5* (ABA Insensitive 5) mutant in rapeseed (*Brassica napus*) that exhibited higher germination rates and more-developed root systems under drought stress. Transcriptomic analysis across multiple timepoints and treatments revealed that *abi5* mutation resulted in more downregulated genes than upregulated ones, indicating a negative regulatory role in gene expression. WGCNA identified glutathione metabolism pathway genes as potential candidates in *ABI5*-regulated drought responses, providing a foundation for improving drought tolerance in rapeseed. Lv et al. [35] identified and characterized a rice high-temperature-sensitive 2 (*hts2*) mutant, revealing that HTS2 encodes CYP71P1, a cytochrome P450 protein involved in serotonin biosynthesis. The hts2 mutant exhibited reduced antioxidant capacity and excessive ROS accumulation under heat stress. Exogenous serotonin supplementation completely rescued the heat-sensitive phenotype. *HTS2* mutation also depressed heat-induced expression of *OsHsfA2s* and downstream targets including *OsHSP18.0* and *OsAPX2*, linking serotonin metabolism to heat shock responses.

This Special Issue also includes four review papers. Kamanga et al. [36] provide a metabolite-centered review of cold stress responses and adaptation mechanisms in *Moringa oleifera*. While moringa’s resilience in tropical climates is well documented, there is little information on its adaptation to prolonged cold stress. This review synthesizes current knowledge on cold-stress adaptation mechanisms and highlights the need for integrative metabolite profiling and identification of cold-tolerant provenances to expand moringa cultivation into temperate regions. Tang et al. [15] reviewed the molecular evolution of copper transporters (COPTs) and transcription factors in plant responses to copper stress, discussing the roles of COPT transporters in Cu uptake, transport, and detoxification, as well as the involvement of organic acid exudation, cell wall binding, vacuolar sequestration, and antioxidant enzymes in Cu homeostasis. Tissue-specific expression analyses revealed distinct spatial regulation of *COPT* genes in roots and leaves, highlighting primary sites of Cu transport and detoxification. eTong et al. [37] systematically reviewed the evolutionary and structural analysis of aquaporin gene families in rice. They summarized the evolutionary origins, structural characteristics, and spatiotemporal expression patterns of rice *AQPs* under physiological and stress conditions. The review discusses the potential of precision gene editing and multi-omics integration to elucidate relationships between AQP evolution, environmental adaptability, and functional specialization. Furthermore, Qin et al. [38] conducted a bibliometric analysis of apoplastic barrier research from 2003 to 2023, analyzing 642 publications using CiteSpace and VOSviewer. Their analysis identified research hotspots, including the genes and hormones regulating apoplastic barrier formation, the influence of stress on apoplastic barriers, and the chemical components of these barriers. Future research directions were proposed, including the physiological functions of apoplastic barriers in roots, differences in barrier formation among root types, methods of promoting barrier formation, and the application of molecular biology techniques.

## 2. Conclusions and Outlook

This Special Issue improves our understanding of crop stress physiology and cellular adaptation mechanisms. Several papers highlight the importance of epigenetic regulation, post-translational modifications, and protein–protein interactions in fine-tuning stress responses [15,17]. Efforts ranging from genome-wide identification and expression profiling to functional characterization, multi-omics integration, and bibliometric analysis show the complexity and sophistication of plant stress responses [39]. Moreover, phytohormone signaling [40], ROS, transcriptional reprogramming, and metabolic adjustment are shown to operate in concert to maintain cellular homeostasis under diverse abiotic stresses. In the future, modern biotechnological tools such as CRISPR/Cas9 (e.g., prime editing and large-fragment insertion) [41], overexpression and RNAi approaches, multi-omics integration (e.g., single-cell and spatial omics) [42], and protoplast transient expression systems will potentiate molecular approaches’ capacity to reveal stress mechanisms and develop stress-tolerant crops [43]. In addition, pangenomes are emerging as valuable resources for exploring genetic variation [44], with pangenomic datasets now available for several plants species [45]. Nevertheless, the contribution of genes associated with improved yield under greenhouse conditions remains to be validated under field conditions [46].

We thank all the authors for their valuable contributions to this Special Issue. Their rigorous research and insightful reviews have improved our understanding of plant stress physiology, providing new perspectives for enhancing crop resilience. We also acknowledge the peer reviewers and the editorial staff for their dedicated efforts in ensuring the quality of these publications. We hope researchers will find these papers both informative and inspiring with respect to their own endeavors in this critically important area.
